# Genotype-Based Gene Expression in Colon Tissue—Prediction Accuracy and Relationship with the Prognosis of Colorectal Cancer Patients

**DOI:** 10.3390/ijms21218150

**Published:** 2020-10-31

**Authors:** Heike Deutelmoser, Justo Lorenzo Bermejo, Axel Benner, Korbinian Weigl, Hanla A. Park, Mariam Haffa, Esther Herpel, Martin Schneider, Cornelia M. Ulrich, Michael Hoffmeister, Jenny Chang-Claude, Hermann Brenner, Dominique Scherer

**Affiliations:** 1 Division of Preventive Oncology, German Cancer Research Center (DKFZ) and National Center for Tumor Diseases (NCT), Im Neuenheimer Feld 460, 69120 Heidelberg, Germany; heike.deutelmoser@nct-heidelberg.de (H.D.); mariam.haffa@nct-heidelberg.de (M.H.); neli.ulrich@hci.utah.edu (C.M.U.); h.brenner@dkfz-heidelberg.de (H.B.); 2 Institute of Medical Biometry and Informatics, Medical Faculty, Heidelberg University, Im Neuenheimer Feld 130.3, 69120 Heidelberg, Germany; lorenzo@imbi.uni-heidelberg.de; 3 Division of Biostatistics, German Cancer Research Center (DKFZ), Im Neuenheimer Feld 581, 69121 Heidelberg, Germany; benner@dkfz-heidelberg.de; 4 Division of Clinical Epidemiology and Aging Research, German Cancer Research Center (DKFZ), Im Neuenheimer Feld 581, 69121 Heidelberg, Germany; k.weigl@dkfz-heidelberg.de (K.W.); m.hoffmeister@dkfz-heidelberg.de (M.H.); 5 Division of Cancer Epidemiology, German Cancer Research Center (DKFZ), Im Neuenheimer Feld 581, 69121 Heidelberg, Germany; h.park@dkfz-heidelberg.de (H.A.P.); j.chang-claude@dkfz-heidelberg.de (J.C.-C.); 6 Division of Translational Functional Cancer Genomics, National Center for Tumor Diseases (NCT) and German Cancer Research Center (DKFZ), Im Neuenheimer Feld 460, 69120 Heidelberg, Germany; 7 NCT Tissue Bank, National Center for Tumor Diseases (NCT) and University Hospital Heidelberg, Im Neuenheimer Feld 460, 69120 Heidelberg, Germany; esther.herpel@med.uni-heidelberg.de; 8 Institute of Pathology, University Hospital Heidelberg, Im Neuenheimer Feld 224, 69120 Heidelberg, Germany; 9 Department of General, Visceral, and Transplantation Surgery, University Hospital Heidelberg, Im Neuenheimer Feld 420, 69120 Heidelberg, Germany; martin.schneider@med.uni-heidelberg.de; 10 Huntsman Cancer Institute, 2000 Cir of Hope Dr 1950, Salt Lake City, UT 84112, USA; 11 Department of Population Health Sciences, School of Medicine, University of Utah, Salt Lake City, UT 84112, USA; 12 Cancer Epidemiology Group, University Cancer Center Hamburg (UCCH), University Medical Center Hamburg-Eppendorf (UKE), Martinstraße 52, 20246 Hamburg, Germany; 13 German Cancer Consortium (DKTK), German Cancer Research Center (DKFZ), Im Neuenheimer Feld 280, 69120 Heidelberg, Germany

**Keywords:** genotype-based gene expression, PrediXcan, colorectal cancer, survival

## Abstract

Colorectal cancer (CRC) survival has environmental and inherited components. The expression of specific genes can be inferred based on individual genotypes—so called expression quantitative trait loci. In this study, we used the PrediXcan method to predict gene expression in normal colon tissue using individual genotype data from 91 CRC patients and examined the correlation ρ between predicted and measured gene expression levels. Out of 5434 predicted genes, 58% showed a negative ρ value and only 16% presented a ρ higher than 0.10. We subsequently investigated the association between genotype-based gene expression in colon tissue for genes with ρ > 0.10 and survival of 4436 CRC patients. We identified an inverse association between the predicted expression of *ARID3B* and CRC-specific survival for patients with a body mass index greater than or equal to 30 kg/m^2^ (HR (hazard ratio) = 0.66 for an expression higher vs. lower than the median, *p* = 0.005). This association was validated using genotype and clinical data from the UK Biobank (HR = 0.74, *p* = 0.04). In addition to the identification of *ARID3B* expression in normal colon tissue as a candidate prognostic biomarker for obese CRC patients, our study illustrates the challenges of genotype-based prediction of gene expression, and the advantage of reassessing the prediction accuracy in a subset of the study population using measured gene expression data.

## 1. Introduction

Colorectal cancer (CRC) is a leading cause of cancer death worldwide [1,2]. Modifiable factors of colorectal cancer patients’ survival include smoking, alcohol consumption, aspirin use, and physical activity, while the effect of obesity is still controversial [3]. In addition, several studies have identified genetic polymorphisms associated with colorectal cancer prognosis [4,5,6,7,8]. The effect of prognostic genetic variants is thought to be, to a large extent, of a regulatory nature, leading to a modulation of the expression of target genes. Single nucleotide polymorphisms (SNP) that modulate gene expression are called expression quantitative trait loci (eQTLs) and may act in cis (modulating the expression of a near-by gene) or in trans (modulating the expression of a distant gene) [9]. Within recent years, immense efforts have been undertaken to map tissue-specific regulatory variants of the human genome [10,11], resulting in a large variety of tools and databases that facilitate the functional characterization of polymorphisms and their proxies identified in genetic association studies [12,13].

The information contained in such databases is the basis of PrediXcan, a method that enables the prediction of tissue-specific gene expression based on individual genotype data [14]. PrediXcan estimates the fraction of genetically determined gene expression levels and performs association analyses between predicted gene expression profiles and a phenotype of interest. This approach potentially accelerates the identification of phenotype-shaping genes. Genome-wide association studies have identified thousands of loci associated with complex traits [15]. The use of PrediXcan is increasingly common in genetic association studies [16,17,18,19,20,21,22,23,24] and recently contributed to the identification of genes associated with lipid levels and schizophrenia [19,20], cutaneous squamous cell carcinoma [23], lung cancer [24], and colorectal cancer [21]. For example, *TRIM4* and *PYGL*, both related to cellular metabolic programming, were associated with CRC risk [21]. However, studies have also reported that the prediction accuracy may be impaired due to, for example, population stratification [25,26]. A limitation of previous studies is that they fully relied on predicted gene expression without consideration of potential differences in prediction accuracy among human populations. Furthermore, to our knowledge, no previous study has used PrediXcan to investigate colorectal cancer prognosis. We thus measured global gene expression profiles in healthy colon mucosa of 91 colorectal cancer patients from the ColoCare-“Darmkrebs: Chancen der Verhuetung durch Screening” (DACHS) study and subsequently inferred gene expression profiles based on individual genotype data using PrediXcan. We calculated the Spearman correlation ρ between the measured and the genetically predicted gene expression levels as a measure of prediction accuracy, and further investigated the association between the genetically-determined gene expression and survival of 4436 colorectal cancer patients for 863 well-predicted genes (ρ > 0.10).

## 2. Results

### 2.1. Correlation between Measured and Genetically Predicted Gene Expression

**Figure 1:**
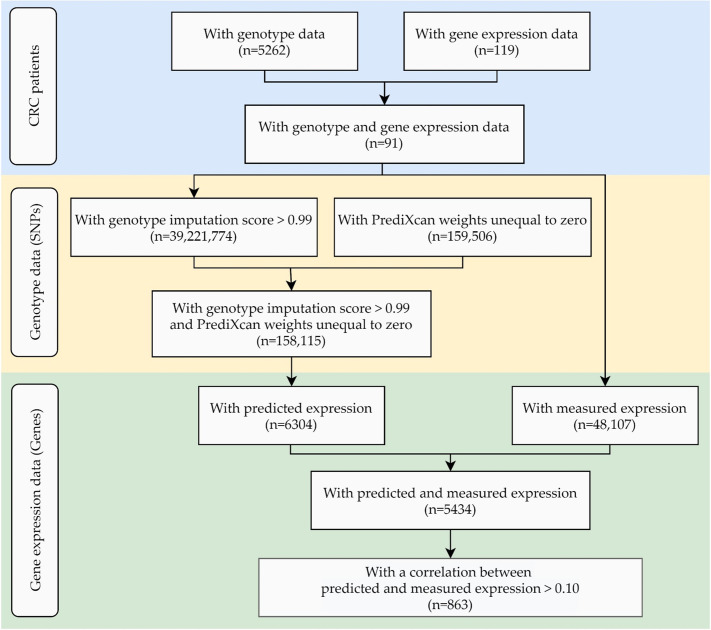
Data overview for the examination of the correlation ρ between predicted and measured gene expression. Abbreviations: CRC: colorectal cancer; SNPs: single nucleotide polymorphisms.

We first examined the correlation between measured and genetically predicted gene expression levels in a subset of 91 colorectal cancer patients, for which genome-wide genotype and gene expression data of healthy colorectal mucosa were available (Figure 1). The characteristics of this subset of the total study population are presented in Table S1.

**Figure 2:**
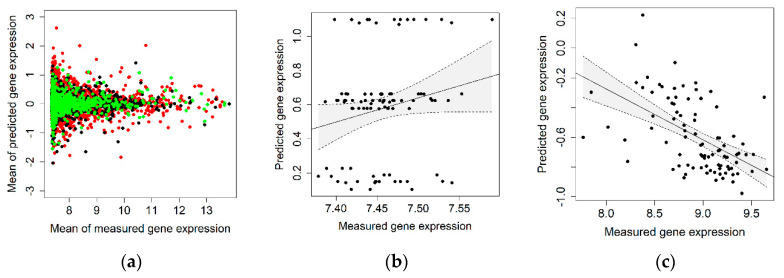
(**a**) Scatter plot of the mean measured versus the mean predicted expression values for 5434 genes with estimated Spearman correlation coefficients ρ < 0 in red, 0 ≤ ρ ≤ 0.1 in black, and ρ > 0.1 in green; (**b**,**c**) measured versus predicted expression values for the genes *TRIM4* (**b**), and *PYGL* (**c**). The linear regression lines are also shown with their corresponding 95% confidence bands.

Out of 159,506 SNPs that PrediXcan uses to predict gene expression in colon transverse tissue, 158,115 SNPs with a genotype imputation score higher than 0.99 were available in our study of 91 participants and were used for the prediction of gene expression in normal colon tissue. This translated into the estimation of gene expression levels of 6304 genes, while measured gene expression data in normal tissue was available for 5434 genes. Figure 2a shows the mean measured (x-axis) versus the mean predicted (y-axis) expression values for the 5434 investigated genes.

The correlation between measured and genetically predicted gene expression among the 91 investigated individuals was negative for 58% of the genes (displayed in red) and between 0 and 0.10 for 26% of the genes (displayed in black), and only 16% (863 genes) presented a correlation higher than 0.10 (displayed in green, listed in Table S2).

PrediXcan has been previously applied to identify novel colorectal cancer risk loci [21,22]. Since the preceding studies included a part of the study population presented here, we investigated the accuracy of the predicted gene expression levels for the previously identified genes. These previous studies reported that the expression of *TRIM4* and *PYGL* in colon transverse tissue was associated with colorectal cancer risk [21], while *PTPN2* expression in colon transverse tissue modified the association of diabetes with colorectal cancer risk [22]. The expression of *PTPN2* was not measured in our study of 91 participants and could thus not be further investigated. However, we examined the correlation between measured and predicted gene expression for *TRIM4* and *PYGL* (Figure 2b-c) and observed a correlation of ϱ = 0.19 for *TRIM4* and a negative correlation of ϱ = −0.56 for *PYGL*. The SNPs as well as the corresponding regression coefficients used to predict the gene expression of *TRIM4* and *PYGL* are provided in Table S3.

### 2.2. Association of Genetically Predicted Gene Expression and Colorectal Cancer Patients’ Survival

We then investigated the association between the genotype-based gene expression for genes with a good prediction accuracy (863 genes with a correlation higher than ϱ = 0.10) and survival of 4436 colorectal cancer patients. Characteristics of the study population for the investigated endpoints overall survival (OS) and disease-specific survival (DSS) are presented in Table 1.

During a median follow up of 6.94 years, 1790 patients died and 1053 died of colorectal cancer. Patients who died from any cause (overall survival) were older, diagnosed at higher stage of the disease, were more likely to be also affected with diabetes, and were less likely to consume alcohol or to be overweight or obese.

The genetically predicted expression of 36 genes was associated with overall survival and of 48 genes with disease-specific survival (Table S4). After adjustment for multiple testing, the smallest probability value was found for the association between the genetically predicted expression of *MAP1B* and disease-specific survival (raw *p* = 0.0002, multiplicity-adjusted *p* = 0.15).

We further performed stratified analyses. Since body mass index (BMI) was the only variable associated with overall and disease-specific survival, which was also available in the UK Biobank, we classified colorectal cancer patients into four BMI groups: BMI < 18.5 kg/m^2^ = underweight, BMI 18.5–24.9 kg/m^2^ = normal weight, BMI 25–29.9 kg/m^2^ = overweight, and BMI ≥ 30 kg/m^2^ = obese, and investigated the association between genetically determined gene expression and survival of colorectal cancer patients in each BMI category.

The expression of more than thirty genes was associated with overall or disease-specific survival of colorectal cancer patients in each BMI group (Table S4). After adjustment for multiple testing, the genetically predicted expression of *ARID3B* showed the strongest association with disease-specific survival (raw *p* = 0.00008, multiplicity-adjusted *p* = 0.07).

**Figure 3:**
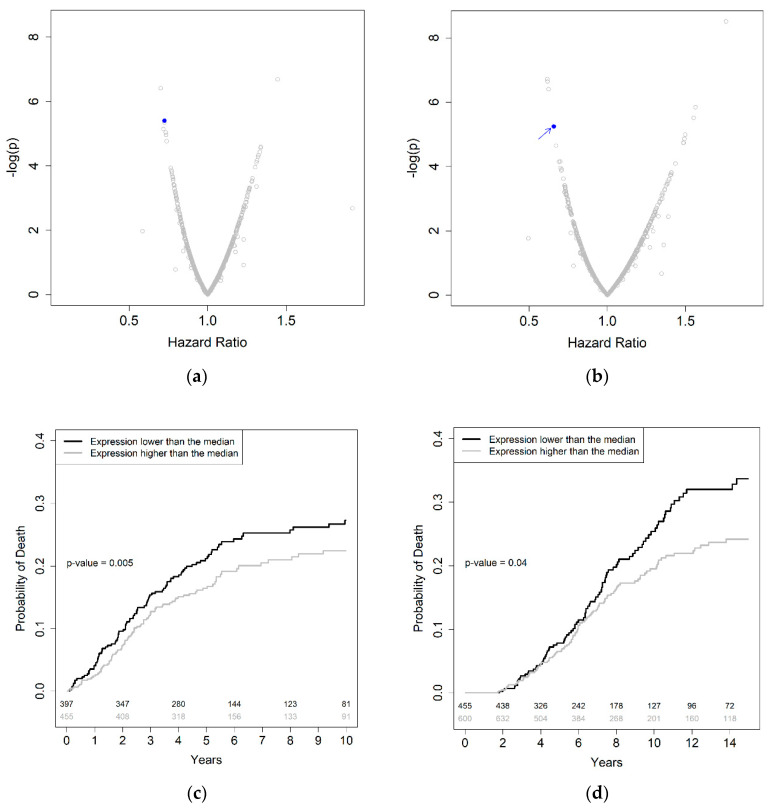
(**a**,**b**) Volcano plots showing the results from survival analyses of 852 CRC patients with a BMI ≥ 30, and 863 genes with a correlation between predicted and measured expression values higher than 0.10. The blue dots indicate results for the gene *ARID3B*. (**a**) Overall survival; (**b**) CRC-specific survival; (**c**,**d**) cumulative probability of death due to CRC for patients with a low (lower than the median) and high (higher than the median) *ARID3B* expression based on individual genotypes for patients with BMI ≥ 30. The number of CRC patients at risk is shown in the lower part of each panel; (**c**) Aalen-Johansen probability curves in the identification cohort (“Darmkrebs: Chancen der Verhuetung durch Screening” (DACHS) study); (**d**) Aalen–Johansen probability curves in the validation cohort (UK Biobank). Abbreviations: CRC: colorectal cancer.

Figure 3 shows the volcano plots of the survival analyses of 852 obese CRC patients in the DACHS study (Figure 3a: overall survival, Figure 3b: disease-specific survival) with the blue dots indicating the results for the gene *ARID3B*. The correlation between the measured and the genotype-based *ARID3B* expression was ϱ = 0.11.

We were further able to validate this association in an independent dataset of 1115 colorectal cancer patients with a BMI higher than or equal to 30 kg/m^2^ in the UK Biobank (*p* = 0.02). The characteristics of the total study population of the validation dataset are presented in Table S5 (median follow up of 7.02 years; 1035 deaths, 669 deaths due to CRC).

Figure 3c,d depicts the disease-specific survival of obese colorectal cancer patients according to predicted expression of *ARID3B* in the discovery (Figure 3c, HR (hazard ratio) = 0.66; *p* = 0.005 for an expression higher vs. lower than the median) and validation datasets (Figure 3d, HR = 0.74; *p* = 0.04). In both the identification and the validation datasets, only obese CRC patients showed an association between genetically predicted *ARID3B* expression and disease-specific survival (Figure S1).

## 3. Discussion

In the present study, we used PrediXcan to infer gene expression in normal colon tissue from 91 colorectal cancer patients and examined the prediction accuracy by comparing the predicted and the measured gene expression levels. We then investigated the association between gene expression for well-predicted genes (ϱ > 0.10) and survival of 4436 colorectal cancer patients from the DACHS study. We observed that out of 5434 genes, for which measured as well as predicted gene expression data were available, 58% showed a negative correlation, while only 16% presented a correlation higher than 0.10 and were thus taken forward to perform association analyses with the survival of colorectal cancer patients.

The PrediXcan prediction model uses a median number of 23 SNPs to predict gene expression (minimum = 1, maximum = 259 SNPs). The expression of *PYGL* was predicted based on seven SNPs, which should suffice to accurately predict gene expression. The negative correlation between measured and predicted expression of *PYGL* (ϱ = −0.58) in our study is unlikely the result of an insufficient number of eQTLs as previous studies reported that genetically regulated gene expression seems to be associated with a small number of variants rather than with multiple eQTLs [27,28]. Nevertheless, the expression of better predicted gene *TRIM4* (ϱ = 0.19) was based on 26 genetic variants.

Negative correlations between measured and genetically predicted gene expression using PrediXcan have been reported in previous studies [25,26] and could also result from weak associations between SNPs and the expression levels of the target gene. Finally, PrediXcan prediction models were trained based on local SNPs (*cis*-eQTLs) within one megabase (MB) of the start or the end of the gene, and the inclusion of potential *trans*-eQTLs could improve the prediction of gene expression.

Recent studies reported that gene expression prediction accuracy varies between populations [25,26]. The reference datasets, which were used to train the PrediXcan prediction models, are the Depression Genes and Networks (DGN) study and the Genotype-Tissue Expression (GTEx) project. The majority of the subjects in these reference datasets are of European descent [10]. Thus, it is not surprising that PrediXcan predicts gene expression in individuals of European descent more accurately than individuals of African descent [26]. However, differences in prediction accuracy were also reported among closely related European populations [26]. Our data corroborates this observation in showing that the correlation between measured and genetically predicted gene expression within Europeans strongly varies for some genes. *PYGL*, which was previously associated with colorectal cancer risk, showed a correlation of 0.51 between measured and predicted gene expression based on data from GTEx [21]. In contrast, in our data we observed a strong negative correlation between measured and predicted gene expression for *PYGL* (ϱ = −0.58), implying that subpopulation differences may also be present in Europeans and need to be considered in genetic prediction tools [26,29]. Alternatively, we propose to measure gene expression in a subset of the total study population to assure that the correlation between predicted and measured gene expression levels of the investigated genes is greater than a preset threshold for the correlation (here 0.10) and to filter for well-predicted genes.

After we filtered for well-predicted genes, we identified one gene that was associated with survival of colorectal cancer patients and was subsequently validated in an independent dataset using genotype and clinical data from the UK Biobank. An increased expression of *ARID3B* was associated with a better disease-specific survival of obese colorectal cancer patients. *ARID3B* (AT-Rich Interaction Domain 3B) encodes for a DNA binding protein and has been described as contributor to tumor initiation and progression in cancerous diseases [30]. A recent study has described the role of ARID3B in colorectal tumor growth [31]. ARID3B has been further described as an oncoprotein and is involved in the progression of malignant neuroblastoma, ovarian cancer, and breast cancer [32,33,34,35]. However, to our knowledge no association of *ARID3B* with overweight or obesity has been reported, and it is unclear why this association was restricted to obese patients and not observed in other BMI-groups.

The present study is based on 4434 colorectal cancer cases with detailed clinical, demographic, and genome-wide genotype data, as well as with global gene expression data for a subset of 91 patients. The availability of paired genotype and gene expression data for a subset of patients enabled us to investigate the correlation between measured and genetically predicted gene expression, which is a major strength of this study. We were thus able to filter for well-predicted genes within our population to subsequently perform association analyses. Furthermore, we had access to an independent dataset from the UK Biobank, in which we validated the association between *ARID3B* expression and disease-specific survival of obese colorectal cancer patients. Although our sample size was fairly large, some of the subgroup analyses were based on small strata and this hampered stratified correlation analyses between measured and predicted gene expression. Furthermore, we did not have access to measured gene expression data from the validation dataset to test for the accuracy of the predicted expression of *ARID3B*.

In conclusion, this study illustrates the challenges of gene expression prediction in normal tissue based on individual genotype data and underlines the importance of assessing prediction accuracy through measuring gene expression in a subset of the investigated study population. Finally, we identified *ARID3B* as a potential survival-modifier in obese colorectal cancer patients.

## 4. Materials and Methods

### 4.1. Study Population

The study population included patients with CRC who participated in a long-term follow-up study of patients of the German population-based case-control DACHS study (“Darmkrebs: Chancen der Verhuetung durch Screening”) [36,37]. CRC patients with a primary, confirmed diagnosis of CRC had been recruited from hospitals of the Rhein-Neckar-Odenwald region since January 2003. Included were patients aged 30 years or older, German speaking, resident in the study region, and mentally and physically able to complete an in-person interview. Baseline standardized questionnaires contained demographic information and information on established or suggested CRC risk factors, as well as possible prognostic factors. Follow-up information on overall and disease-specific survival was collected at three, five, and ten years after diagnosis. Causes of death were verified by death certificates and coded based on ICD-10 classifications. Information on recurrences were collected from general practitioners and specialists as applicable. In addition, clinical data was extracted from patient records. Population controls were randomly selected from lists of residents of the population registries of the cities and counties. The study was approved by the ethics committee (approved on 6 December 2001, project identification code 310/2001) of the University of Heidelberg and State Medical Boards of Baden-Wuerttemberg and was conducted in agreement with the Helsinki Declaration. Written informed consent was provided by all participants at baseline and during follow-up.

### 4.2. Genotyping

5262 DACHS samples were genotyped using the whole-genome Illumina CytoSNP assay (Illumina, San Diego, CA, USA) for patients recruited 2003–2007, the Illumina HumanOmniExpress BeadChip Kit for patients recruited 2008–2014, and the Illumina Infinium OncoArray-500K BeadChip for those recruited 2015 or the Infinium Global Screening Array for patients recruited 2016–2017 as described previously [38]. Missing genotypes were imputed using the Haplotype Reference Consortium as reference panel l (HRC r1.1 2016).

### 4.3. Gene Expression Measurement

Gene expression profiles of healthy colorectal mucosa tissues from 91 participants of a subsample of the DACHS study (ColoCare-DACHS study) were measured using Illumina HumanHT-12 Expression BeadChips according to the manufacturer’s instructions as described previously [39,40]. Raw gene expression data was processed prior to statistical analyses. Missing expression values were imputed using the nearest neighbor averaging method as implemented in the R package impute. Expression data were adjusted for batch effects using the R package “sva” and subsequently transformed using the variance stabilizing transformation method and normalized using the robust spline normalization method of the R package “lumi”.

### 4.4. Gene Expression Prediction

Out of the 4465 CRC patients of the DACHS study with available genome-wide genotype and follow-up data, we used 4436 CRC patients with available information on age, gender, stage at diagnosis and tumor site, and a minimum follow-up of 30 days.

DACHS genotype data was combined and quality controlled using the info score of the program qctool (https://www.well.ox.ac.uk/~gav/qctool_v2). PrediXcan transcriptome prediction models were downloaded from the publicly available PredictDB repository (www.predictdb.org). Gene expression profiles in colon normal tissue were estimated based on genome-wide genotype data of DACHS patients using the software PrediXcan (https://github.com/hakyimlab/PrediXcan) [14]. PrediXcan provides prediction models trained by elastic net models and using reference datasets from the Genotype-Tissue Expression Project (GTEx), where the majority of subjects are of European descent. The reference dataset for normal colon tissue contained 169 colon transverse samples resulting in 159,506 variants with a prediction weight unequal to zero and used for the gene expression prediction of 6304 colon genes in normal tissue [10].

As the proportion of genetically determined gene expression levels may differ by population, we further tested the accuracy of predicted gene expression levels in the DACHS study. Observed gene expression profiles in healthy colorectal mucosa tissues of 91 CRC patients from the ColoCare-DACHS study were correlated with the predicted gene expression profiles.

Genes with available measured gene expression and with a median absolute deviation (MAD) of measured and predicted gene expression greater than 0 were included. For each gene, a quality metric ρ was computed as the Spearman correlation between the observed and predicted expression. Association tests were restricted to genes with a predictive ϱ ≥ 0.10, i.e., ≥10% correlation between predicted and observed expression (cf. [21,22]).

### 4.5. Statistical Analysis

We tested the association between genetically determined expression levels in healthy colon tissue and the survival of 4436 CRC patients from the DACHS study in 863 genes with a predictive ϱ ≥ 0.10. Cox proportional hazards regression was performed to test the predicted gene expression associations with overall survival and disease-specific survival. Survival time was calculated from date of diagnosis until date of death by any cause for overall survival and from date of diagnosis until date of death by CRC for disease-specific survival. Age, sex, stage at diagnosis, and cancer site were included in the model as relevant prognostic factors. Individuals with missing entries were excluded. The proportional hazards assumption was tested according to Grambsch and Therneau for a set of variables including age (<60 years, 60–69 years, 70–79 years, ≥80 years), sex, stage at diagnosis (I, II, III, IV), cancer site (colon, rectum), chemotherapy (yes, no), diabetes (yes, no), BMI (<18.5, 18.5–24.9, 25–29.9, ≥30 kg/m^2^), regular use of non-steroidal anti-inflammatory drugs (NSAIDs) more than twice per week for at least one year (yes, no), regular smoking (never, former, and current) and alcohol intake (0, 0–5.6, 5.7–13.2, 13.3–28.5, and ≥28.6 g/day). Survival analyses were evaluated using as predictor variables the predicted gene expression levels as continuous variables and subsequently the predicted gene expression levels dichotomized at the median level for Cox proportional hazard models to estimate hazard ratios (HR) for overall survival and disease-specific survival, and their 95% confidence intervals (CIs). The median follow-up time and the cumulative probability of death were calculated using the Aalen–Johansen estimator. Bonferroni–Holm correction method was used to adjust for multiple testing.

The statistical analysis was carried out using SAS version 9.3 (SAS Institute, Cary, NC, USA) and R version 3.1.0 (www.r-project.org).

### 4.6. Validation Set

Validation of associations was performed using the UK Biobank resource. UK Biobank recruited 500,000 people from the UK aged between 40 and 69 years in 2006–2010 [41]. Genotype calling was performed by Affymetrix on the UK BiLEVE Axiom array and the UK Biobank Axiom array (Affymetrix, Santa Clara, CA, USA). Genotypes were imputed using the Haplotype Reference Consortium and UK10K haplotype resources [42].

The validation set included 4241 CRC patients from the UK Biobank resource (Table S5). Statistical analyses from our respective findings were performed as described above. Analyses regarding the registered endpoints overall and disease-specific survival were adjusted for the available variables age and sex. Subgroup analyses for the variable BMI were performed.

## Figures and Tables

**Table 1:** Characteristics of the study population.

			Overall Survival			CRC-Specific Survival		
Variable	Level	Patients	Deaths	HR ^1^ (95% CI)	*p*	Deaths	HR ^1^ (95% CI)	*p*
Age at	<60	946	257	Ref.	<2 × 10^−16^	209	Ref.	0.0003
Diagnosis	60–69	1339	466	1.25 (1.07−1.46) 1.69 (1.47−1.96) 3.20 (2.74−3.74)		314	1.06 (0.89−1.27) 1.12 (0.96−1.36) 1.47 (1.20−1.80)	
(years)	70–79	1489	638			357		
	>80	662	429			173		
Gender	Male	2685	1111	Ref.	0.16	626	Ref.	0.47
	Female	1751	679	1.07 (0.97−1.18)		427	0.96 (0.85−1.08)	
CRC stage	I	1024	235	Ref.	<2 × 10^−16^	40	Ref.	<2 × 10^−16^
	II	1345	434	1.49 (1.27−1.74) 2.05 (1.76−2.39) 10.21 (8.73−11.94)		148	2.96 (2.09−4.20) 7.29 (5.25−10.10) 48.91 (35.37−67.64)	
	III	1426	575			354		
	IV	641	546			511		
Tumor site	Colon	2665	1069	Ref.	0.61	598	Ref.	0.03
	Rectum	1771	721	1.03 (0.93–1.13)		455	1.15 (1.02−1.30)	
Body mass	<18.5	121	71	1.74 (1.37−2.23)	0.0006	41	1.62 (1.17−2.23)	0.01
index	18.5−24.9	1592	675	Ref.		396	Ref.	
(kg/m^2^)	25–29.9	1871	726	0.87 (0.78−0.96) 0.83 (0.73−0.95)		432	0.88 (0.77−1.01) 0.83 (0.70–0.99)	
	≥30	852	318			184		
Diabetes	No	3594	1360	Ref.	<8 × 10^−13^	844	Ref.	0.09
	Yes	811	412	1.50 (1.34−1.67)		200	1.14 (0.98−1.33)	
Regular	No	3272	1298	Ref.	0.05	793	Ref.	0.31
NSAID use	Yes	1105	469	1.11 (1.00−1.24)		245	0.93 (0.80−1.07)	
Smoking	Never	1921	840	Ref.	0.04	515	Ref.	0.07
	Former	1769	684	0.90 (0.81−1.00) 0.89 (0.78−1.03)		367	0.79 (0.69−0.90) 0.94 (0.79−1.12)	
	Current	672	263			169		
Alcohol	No intake	1320	606	Ref.	0.0003	339	Ref.	0.01
intake	0.1−5.6	856	318	0.75 (0.66−0.86) 0.72 (0.63−0.83) 0.78 (0.68−0.90) 0.79 (0.69−0.92)		202	0.87 (0.73−1.03) 1.22 (0.68−0.98) 1.20 (0.70−1.00) 1.24 (0.66−0.97)	
(g/day)	5.7−13.2	770	280			175		
	13.3−28.5	775	301			177		
	≥28.6	669	269			148		

^1^ Hazard ratios adjusted for age, sex, CRC stage, and tumor site. Abbreviations: HR: hazard ratio; CI: confidence interval; CRC: colorectal cancer; Ref.: reference category; NSAID: non-steroidal anti-inflammatory drug.

## Supplementary Materials

Supplementary materials can be found at https://www.mdpi.com/1422-0067/21/21/8150/s1. Table S1: Characteristics of the 91 colorectal cancer patients with measured gene expression data in normal tissue, Table S2: List of 863 well-predicted genes (ϱ > 0.10), Table S3: SNPs and their corresponding weights used for the prediction of the genes TRIM4 and PYGL (extracted from the PrediXcan transverse colon prediction model), Table S4a: *p*-values from the Cox regression for the association between the continuous predicted gene expression and survival for overall survival (OS), Table S4b: *p*-values from the Cox regression for the association between the continuous predicted gene expression and disease-specific survival (DSS), Table S5: Characteristics of the 4241 colorectal cancer patients from the validation set, Figure S1: Probability of death due to CRC for patients with a low (lower than the median) and high (higher than the median) ARID3B expression based on individual genotypes. The number of CRC patients at risk is shown in the lower part of each panel; (a,b) Aalen-Johansen curves in the identification cohort in patients with (a) a BMI between 18.5–24.9 (b) a BMI between 25–29.9; (c,d) Aalen-Johansen curves in the validation cohort in patients with (c) a BMI between 18.5–24.9 (d) a BMI between 25–29.9. Aalen-Johansen curves of patients with a BMI < 18.5 are not shown due to low sample size.
