# Supplementary material for: Genotype-Based Gene Expression in Colon Tissue—Prediction Accuracy and Relationship with the Prognosis of Colorectal Cancer Patients
Source: Int J Mol Sci. 2020 Oct 31;21(21):8150. doi: 10.3390/ijms21218150 (PMC7662650; doi:10.3390/ijms21218150)
Supplement: Supplementary file 1 [file ijms-21-08150-s001.zip › Supplementary Material/TableS1.docx]

**Table S1.** Characteristics of the 91 colorectal cancer patients with measured gene expression data in normal tissue.

| **Variable** | **Level** | **Patients** |
| --- | --- | --- |
| Age at diagnosis (years) | < 60 | 31 |
|  | 60 – 69 | 28 |
|  | 70 – 79 | 27 |
|  | > 80 | 5 |
| Gender | Male | 57 |
|  | Female | 34 |
| CRC stage | I | 17 |
|  | II | 31 |
|  | III | 29 |
|  | IV | 13 |
| Tumor site | Colon | 40 |
|  | Rectum | 51 |
| Body mass index (kg/m^2^) | < 18.5 | 1 |
|  | 18.5-24.9 | 24 |
|  | 25-29.9 | 44 |
|  | ≥ 30 | 22 |
| Diabetes | No | 80 |
|  | Yes | 9 |
| Regular NSAID use | No | 62 |
|  | Yes | 29 |
| Smoking | Never | 33 |
|  | Former | 40 |
|  | Current | 18 |
| Alcohol intake (g/day) | No intake | 10 |
|  | 0.1-5.6 | 21 |
|  | 5.7-13.2 | 19 |
|  | 13.3-28.5 | 21 |
|  | ≥ 28.6 | 20 |

Abbreviations: CRC: colorectal cancer.
