# Supplementary material for: Genotype-Based Gene Expression in Colon Tissue—Prediction Accuracy and Relationship with the Prognosis of Colorectal Cancer Patients
Source: Int J Mol Sci. 2020 Oct 31;21(21):8150. doi: 10.3390/ijms21218150 (PMC7662650; doi:10.3390/ijms21218150)
Supplement: Supplementary file 1 [file ijms-21-08150-s001.zip › Supplementary Material/TableS2.docx]

**Table S2:** List of 863 well-predicted genes (ϱ > 0.10).

| Symbol | Spearman correlation ρ |
| --- | --- |
| *PTER* | 0.79 |
| *HLA_DQB1* | 0.58 |
| *TIMM10* | 0.57 |
| *RIPK5* | 0.57 |
| *KRIT1* | 0.52 |
| *SPG7* | 0.52 |
| *RAD21* | 0.51 |
| *B3GALTL* | 0.49 |
| *BCR* | 0.48 |
| *RPL13* | 0.47 |
| *RPS6KB2* | 0.42 |
| *MIF* | 0.41 |
| *IRF5* | 0.39 |
| *C18orf10* | 0.39 |
| *YWHAB* | 0.38 |
| *PPIE* | 0.37 |
| *MTMR3* | 0.36 |
| *KDELR2* | 0.35 |
| *LEPROT* | 0.35 |
| *HEXIM1* | 0.34 |
| *AGL* | 0.34 |
| *BBS7* | 0.34 |
| *SERF1A* | 0.33 |
| *CRELD1* | 0.33 |
| *LYSMD1* | 0.33 |
| *UBA52* | 0.33 |
| *RECQL5* | 0.32 |
| *LOC729852* | 0.31 |
| *MFSD11* | 0.31 |
| *DIO2* | 0.31 |
| *ANKRD13D* | 0.31 |
| *TMEM128* | 0.31 |
| *ZFYVE21* | 0.30 |
| *ACCN3* | 0.30 |
| *GPATCH4* | 0.30 |
| *GYPE* | 0.30 |
| *SLC25A26* | 0.30 |
| *FXYD2* | 0.29 |
| *FAM160B1* | 0.29 |
| *CC2D1B* | 0.29 |
| *SMC6* | 0.29 |
| *DNAJC13* | 0.29 |
| *PTPN12* | 0.29 |
| *MEST* | 0.29 |
| *ANKMY1* | 0.29 |
| *C6orf142* | 0.28 |
| *CABIN1* | 0.28 |
| *CCDC58* | 0.28 |
| *ZNF417* | 0.28 |
| *GFM1* | 0.28 |
| *ZNF491* | 0.28 |
| *PSAP* | 0.28 |
| *SLC4A5* | 0.28 |
| *WDR73* | 0.27 |
| *C6orf165* | 0.27 |
| *UTP15* | 0.27 |
| *LRDD* | 0.27 |
| *SPPL2B* | 0.27 |
| *GUF1* | 0.27 |
| *DPP10* | 0.27 |
| *ENTPD4* | 0.27 |
| *MCART1* | 0.27 |
| *RNASEN* | 0.26 |
| *ACOX3* | 0.26 |
| *SAP30L* | 0.26 |
| *C15orf38* | 0.26 |
| *RNPS1* | 0.26 |
| *SFI1* | 0.26 |
| *RNF10* | 0.26 |
| *C14orf43* | 0.26 |
| *SRGAP1* | 0.26 |
| *CSE1L* | 0.26 |
| *C9orf43* | 0.26 |
| *C17orf101* | 0.26 |
| *MAPT* | 0.26 |
| *TK2* | 0.26 |
| *ERAP1* | 0.26 |
| *RFTN2* | 0.26 |
| *C1GALT1* | 0.26 |
| *NICN1* | 0.26 |
| *FAM129B* | 0.26 |
| *ZNF721* | 0.25 |
| *F7* | 0.25 |
| *KIAA1430* | 0.25 |
| *KIAA1086* | 0.25 |
| *TM7SF3* | 0.25 |
| *POLR2D* | 0.25 |
| *PPAP2C* | 0.25 |
| *TCP1* | 0.25 |
| *MAGI1* | 0.25 |
| *ASAH2* | 0.25 |
| *THAP7* | 0.25 |
| *LRRC44* | 0.25 |
| *HOXA3* | 0.24 |
| *MSR1* | 0.24 |
| *NFASC* | 0.24 |
| *MBD2* | 0.24 |
| *LEMD3* | 0.24 |
| *F2R* | 0.24 |
| *NLRC3* | 0.24 |
| *SMCHD1* | 0.24 |
| *ANKRD36* | 0.24 |
| *PSMG4* | 0.24 |
| *ZNF626* | 0.24 |
| *SLC26A1* | 0.24 |
| *USP24* | 0.24 |
| *CHID1* | 0.24 |
| *CETP* | 0.24 |
| *C1QTNF6* | 0.24 |
| *ADAM11* | 0.24 |
| *OSBPL9* | 0.24 |
| *SLC19A3* | 0.24 |
| *EPHA5* | 0.24 |
| *ZNF646* | 0.24 |
| *CRMP1* | 0.23 |
| *HS6ST3* | 0.23 |
| *C9orf100* | 0.23 |
| *LILRB2* | 0.23 |
| *LIPC* | 0.23 |
| *WDR90* | 0.23 |
| *PRDX2* | 0.23 |
| *TELO2* | 0.23 |
| *KCNE3* | 0.23 |
| *MRPL17* | 0.23 |
| *PHACTR1* | 0.22 |
| *IZUMO1* | 0.22 |
| *PRDM2* | 0.22 |
| *RAD54L2* | 0.22 |
| *CTNS* | 0.22 |
| *ATG4B* | 0.22 |
| *CPEB1* | 0.22 |
| *FLJ23152* | 0.22 |
| *THBS3* | 0.22 |
| *EPHA3* | 0.22 |
| *VANGL2* | 0.22 |
| *FCAMR* | 0.22 |
| *PDCD6* | 0.22 |
| *PARP10* | 0.22 |
| *ANKRD41* | 0.22 |
| *HSD11B1* | 0.22 |
| *FXYD5* | 0.22 |
| *NFATC3* | 0.22 |
| *DNAH10* | 0.22 |
| *IRGQ* | 0.22 |
| *AOF2* | 0.22 |
| *SIGLEC5* | 0.22 |
| *DENND5B* | 0.22 |
| *KLHL33* | 0.22 |
| *CTSB* | 0.22 |
| *FBXO7* | 0.21 |
| *COQ6* | 0.21 |
| *DTNB* | 0.21 |
| *BEND7* | 0.21 |
| *ZNF66* | 0.21 |
| *TRIM10* | 0.21 |
| *ZNF837* | 0.21 |
| *FKBP7* | 0.21 |
| *CDK5RAP2* | 0.21 |
| *RUVBL2* | 0.21 |
| *NPY1R* | 0.21 |
| *COMMD5* | 0.21 |
| *FLJ39660* | 0.21 |
| *PCBP2* | 0.21 |
| *SDC3* | 0.21 |
| *ALOX5* | 0.21 |
| *SRP14* | 0.21 |
| *MRPL45* | 0.21 |
| *CRHR2* | 0.21 |
| *HLA_F* | 0.21 |
| *EYA2* | 0.21 |
| *UBE2E3* | 0.21 |
| *MUC4* | 0.21 |
| *KIAA1967* | 0.21 |
| *FAM134B* | 0.21 |
| *C13orf23* | 0.21 |
| *SNTG2* | 0.21 |
| *LRRC8C* | 0.21 |
| *KIAA1245* | 0.21 |
| *ZNF438* | 0.21 |
| *RNF14* | 0.21 |
| *PIGG* | 0.20 |
| *RFT1* | 0.20 |
| *C16orf55* | 0.20 |
| *DCHS2* | 0.20 |
| *PARP8* | 0.20 |
| *MDM4* | 0.20 |
| *SHF* | 0.20 |
| *KCNIP3* | 0.20 |
| *TWSG1* | 0.20 |
| *MTRF1L* | 0.20 |
| *EIF4G2* | 0.20 |
| *LIPM* | 0.20 |
| *KCNC4* | 0.20 |
| *PLCL4* | 0.20 |
| *FBF1* | 0.20 |
| *TNKS2* | 0.20 |
| *C2orf34* | 0.20 |
| *HLA_C* | 0.20 |
| *CMTM3* | 0.20 |
| *C17orf57* | 0.20 |
| *PCTK3* | 0.20 |
| *TRIM4* | 0.20 |
| *TMPRSS3* | 0.20 |
| *ZNF597* | 0.20 |
| *CCL14* | 0.20 |
| *CLUAP1* | 0.20 |
| *SORD* | 0.20 |
| *MSH5* | 0.19 |
| *MAP1B* | 0.19 |
| *C6orf52* | 0.19 |
| *ITPR2* | 0.19 |
| *ADNP* | 0.19 |
| *GOSR1* | 0.19 |
| *SNX27* | 0.19 |
| *ST3GAL5* | 0.19 |
| *CKS1B* | 0.19 |
| *KIAA0020* | 0.19 |
| *PCDHGA1* | 0.19 |
| *LYST* | 0.19 |
| *AKAP11* | 0.19 |
| *PLEC1* | 0.19 |
| *AGPAT1* | 0.19 |
| *PDZK3* | 0.19 |
| *PCDH8* | 0.19 |
| *POMGNT1* | 0.19 |
| *KRT39* | 0.19 |
| *POLRMT* | 0.19 |
| *NTRK1* | 0.19 |
| *TMEM80* | 0.19 |
| *MGC40499* | 0.19 |
| *CHRFAM7A* | 0.19 |
| *MED31* | 0.19 |
| *PPFIA1* | 0.19 |
| *PFKL* | 0.19 |
| *NEK3* | 0.19 |
| *LSM7* | 0.19 |
| *CTNNA3* | 0.19 |
| *STON1* | 0.19 |
| *ABCA2* | 0.19 |
| *MCF2L* | 0.19 |
| *UBE2L6* | 0.19 |
| *PPM2C* | 0.18 |
| *LBH* | 0.18 |
| *VPS24* | 0.18 |
| *PPAPDC3* | 0.18 |
| *LOC441869* | 0.18 |
| *DOPEY2* | 0.18 |
| *SLC25A42* | 0.18 |
| *TIGD7* | 0.18 |
| *SIP1* | 0.18 |
| *GAK* | 0.18 |
| *IQCG* | 0.18 |
| *ZNF639* | 0.18 |
| *TRIM63* | 0.18 |
| *ZNF324B* | 0.18 |
| *WWP2* | 0.18 |
| *C21orf33* | 0.18 |
| *GSTM4* | 0.18 |
| *AMPD3* | 0.18 |
| *ALS2CR8* | 0.18 |
| *ATXN10* | 0.18 |
| *DPP8* | 0.18 |
| *IFITM3* | 0.18 |
| *SLC26A8* | 0.18 |
| *FAM86B1* | 0.18 |
| *SAPS2* | 0.18 |
| *VPS41* | 0.18 |
| *OPN4* | 0.18 |
| *MARCH1* | 0.18 |
| *ACTN3* | 0.18 |
| *PGPEP1* | 0.18 |
| *FKBP1A* | 0.18 |
| *C1orf86* | 0.18 |
| *ARHGAP8* | 0.18 |
| *WNT4* | 0.17 |
| *MGLL* | 0.17 |
| *MST1* | 0.17 |
| *GGT1* | 0.17 |
| *NUDT22* | 0.17 |
| *ZNF76* | 0.17 |
| *SLC5A12* | 0.17 |
| *FRMD4A* | 0.17 |
| *APOBEC3H* | 0.17 |
| *FOS* | 0.17 |
| *TTC27* | 0.17 |
| *XYLT2* | 0.17 |
| *CHFR* | 0.17 |
| *C9orf68* | 0.17 |
| *IL1RN* | 0.17 |
| *FUT10* | 0.17 |
| *MEGF9* | 0.17 |
| *SIRT3* | 0.17 |
| *C1orf123* | 0.17 |
| *PCDHA2* | 0.17 |
| *PSCD2* | 0.17 |
| *GNB1L* | 0.17 |
| *C20orf111* | 0.17 |
| *IFNAR2* | 0.17 |
| *INMT* | 0.17 |
| *ARHGEF11* | 0.17 |
| *LRRK2* | 0.17 |
| *ALDH1L2* | 0.17 |
| *GBP7* | 0.17 |
| *PIWIL2* | 0.17 |
| *TMX4* | 0.17 |
| *PHACTR4* | 0.17 |
| *KIAA0194* | 0.17 |
| *CSNK1G2* | 0.17 |
| *C22orf9* | 0.17 |
| *WDR36* | 0.17 |
| *C1orf43* | 0.17 |
| *FUT6* | 0.17 |
| *LFNG* | 0.17 |
| *HDLBP* | 0.17 |
| *SLC45A2* | 0.17 |
| *TMEM50B* | 0.17 |
| *LIG1* | 0.17 |
| *PACRG* | 0.17 |
| *DCHS1* | 0.17 |
| *SDK1* | 0.17 |
| *KIAA0953* | 0.17 |
| *ARPC2* | 0.17 |
| *CHPF* | 0.17 |
| *LSS* | 0.17 |
| *CNTF* | 0.17 |
| *ATF4* | 0.17 |
| *CCDC123* | 0.17 |
| *ODF2* | 0.17 |
| *FLJ20581* | 0.17 |
| *KIAA0564* | 0.17 |
| *ABHD4* | 0.17 |
| *LOC647135* | 0.16 |
| *UBOX5* | 0.16 |
| *C8orf37* | 0.16 |
| *HEATR4* | 0.16 |
| *ZIK1* | 0.16 |
| *CAMK2B* | 0.16 |
| *CLEC2D* | 0.16 |
| *P2RX2* | 0.16 |
| *XIRP1* | 0.16 |
| *ZNF98* | 0.16 |
| *ODAM* | 0.16 |
| *RUVBL1* | 0.16 |
| *CLOCK* | 0.16 |
| *MARCH8* | 0.16 |
| *FOXO6* | 0.16 |
| *CMTM7* | 0.16 |
| *PPP1R1C* | 0.16 |
| *DPY19L2* | 0.16 |
| *HYAL1* | 0.16 |
| *SPEF2* | 0.16 |
| *FAIM* | 0.16 |
| *FAM153A* | 0.16 |
| *CD1D* | 0.16 |
| *BMP2* | 0.16 |
| *NCF4* | 0.16 |
| *CER1* | 0.16 |
| *ASTN2* | 0.16 |
| *SNN* | 0.16 |
| *ZNF441* | 0.16 |
| *POU1F1* | 0.16 |
| *DOCK7* | 0.16 |
| *FIBIN* | 0.16 |
| *LOC100131187* | 0.16 |
| *FLJ10803* | 0.16 |
| *DNAJB12* | 0.16 |
| *KLC1* | 0.16 |
| *AICDA* | 0.16 |
| *SMOX* | 0.16 |
| *ZNF410* | 0.16 |
| *AKT1* | 0.16 |
| *THADA* | 0.16 |
| *FAM40B* | 0.16 |
| *NEK6* | 0.16 |
| *KCNJ14* | 0.16 |
| *GABPB2* | 0.16 |
| *SIRPB2* | 0.16 |
| *SCNN1D* | 0.16 |
| *PPID* | 0.16 |
| *ARHGAP12* | 0.16 |
| *CDC14A* | 0.16 |
| *SPATA2L* | 0.16 |
| *DPP9* | 0.16 |
| *VSTM2B* | 0.16 |
| *MAPK8* | 0.15 |
| *CCNT2* | 0.15 |
| *SIRPD* | 0.15 |
| *RAB24* | 0.15 |
| *PTP4A2* | 0.15 |
| *ZDHHC23* | 0.15 |
| *PTPRE* | 0.15 |
| *RAB4B* | 0.15 |
| *MAP2K5* | 0.15 |
| *RNASE4* | 0.15 |
| *ITGB1BP1* | 0.15 |
| *NPAS3* | 0.15 |
| *TMEM120B* | 0.15 |
| *BTBD9* | 0.15 |
| *ROPN1B* | 0.15 |
| *GNPTAB* | 0.15 |
| *NUCB2* | 0.15 |
| *PLOD1* | 0.15 |
| *C22orf36* | 0.15 |
| *RCN3* | 0.15 |
| *SHC2* | 0.15 |
| *SOAT2* | 0.15 |
| *NPIP* | 0.15 |
| *LOC440836* | 0.15 |
| *FUZ* | 0.15 |
| *TMTC2* | 0.15 |
| *ANXA6* | 0.15 |
| *ELOVL4* | 0.15 |
| *LY6K* | 0.15 |
| *FGFBP3* | 0.15 |
| *TRPV1* | 0.15 |
| *SLC44A1* | 0.15 |
| *CHN1* | 0.15 |
| *NADSYN1* | 0.15 |
| *ATF1* | 0.15 |
| *DDHD2* | 0.15 |
| *PSMA4* | 0.15 |
| *QRICH1* | 0.15 |
| *RUFY2* | 0.15 |
| *SENP5* | 0.15 |
| *HIF3A* | 0.15 |
| *FUT11* | 0.15 |
| *STK3* | 0.15 |
| *AP3M1* | 0.15 |
| *SP110* | 0.15 |
| *C1orf9* | 0.15 |
| *PPAP2A* | 0.15 |
| *PECI* | 0.15 |
| *HNRNPA1L2* | 0.15 |
| *PTPLAD1* | 0.15 |
| *VAMP1* | 0.15 |
| *KIF3A* | 0.15 |
| *MZF1* | 0.15 |
| *MCC* | 0.15 |
| *ATN1* | 0.15 |
| *WBP11* | 0.15 |
| *ING5* | 0.15 |
| *CASP7* | 0.14 |
| *C7orf28A* | 0.14 |
| *GJA4* | 0.14 |
| *ZNHIT1* | 0.14 |
| *MFAP2* | 0.14 |
| *MSH3* | 0.14 |
| *FLCN* | 0.14 |
| *TOP1MT* | 0.14 |
| *XKR6* | 0.14 |
| *PSMC4* | 0.14 |
| *KCNJ10* | 0.14 |
| *LMF2* | 0.14 |
| *AMN* | 0.14 |
| *NDE1* | 0.14 |
| *SURF6* | 0.14 |
| *ACOT7* | 0.14 |
| *HIPK4* | 0.14 |
| *KCNJ4* | 0.14 |
| *PMFBP1* | 0.14 |
| *ZBTB32* | 0.14 |
| *FMNL2* | 0.14 |
| *ARHGAP25* | 0.14 |
| *DHRS4* | 0.14 |
| *MANBAL* | 0.14 |
| *LITAF* | 0.14 |
| *FOXK2* | 0.14 |
| *CRCP* | 0.14 |
| *TAC4* | 0.14 |
| *CLN8* | 0.14 |
| *C10orf6* | 0.14 |
| *RASA1* | 0.14 |
| *USP44* | 0.14 |
| *GRINL1A* | 0.14 |
| *CRIM2* | 0.14 |
| *TACC2* | 0.14 |
| *METT5D1* | 0.14 |
| *NCOA3* | 0.14 |
| *FAM119A* | 0.14 |
| *MAEA* | 0.14 |
| *TRIM73* | 0.14 |
| *DHODH* | 0.14 |
| *KIAA1772* | 0.14 |
| *TGM5* | 0.14 |
| *DHX58* | 0.14 |
| *ZSCAN23* | 0.14 |
| *RABGAP1L* | 0.14 |
| *ATP2A2* | 0.14 |
| *LOC149478* | 0.14 |
| *UBE2I* | 0.14 |
| *C14orf149* | 0.14 |
| *TXNDC3* | 0.14 |
| *ADAMTS7* | 0.14 |
| *EBF2* | 0.14 |
| *APOL3* | 0.14 |
| *C1orf170* | 0.14 |
| *BTNL3* | 0.14 |
| *TREX1* | 0.14 |
| *CYB5R2* | 0.14 |
| *TRIM38* | 0.14 |
| *ZNF575* | 0.14 |
| *CNTN2* | 0.14 |
| *SH3BP5L* | 0.14 |
| *LOC283487* | 0.14 |
| *PCDH21* | 0.14 |
| *VPS29* | 0.14 |
| *GDPD3* | 0.14 |
| *AMD1* | 0.14 |
| *HAPLN4* | 0.14 |
| *KIAA0922* | 0.14 |
| *COPE* | 0.13 |
| *CLCC1* | 0.13 |
| *M6PRBP1* | 0.13 |
| *ACTL8* | 0.13 |
| *SATB1* | 0.13 |
| *REPIN1* | 0.13 |
| *FAM53C* | 0.13 |
| *MTMR10* | 0.13 |
| *CASP8* | 0.13 |
| *CDC2L2* | 0.13 |
| *POL3S* | 0.13 |
| *PBLD* | 0.13 |
| *FNIP2* | 0.13 |
| *LOC123688* | 0.13 |
| *CDH6* | 0.13 |
| *GPIHBP1* | 0.13 |
| *ALKBH6* | 0.13 |
| *NHLRC2* | 0.13 |
| *TXNRD2* | 0.13 |
| *TEX14* | 0.13 |
| *FMO2* | 0.13 |
| *STAP2* | 0.13 |
| *RAB40C* | 0.13 |
| *KCNIP4* | 0.13 |
| *GLIPR1L2* | 0.13 |
| *KLK5* | 0.13 |
| *XCR1* | 0.13 |
| *C14orf109* | 0.13 |
| *ZDHHC11* | 0.13 |
| *PCDHB11* | 0.13 |
| *HMBS* | 0.13 |
| *KIAA0692* | 0.13 |
| *METRN* | 0.13 |
| *LOC653464* | 0.13 |
| *SEPHS1* | 0.13 |
| *BDH2* | 0.13 |
| *PICALM* | 0.13 |
| *CD22* | 0.13 |
| *GLTP* | 0.13 |
| *C1orf83* | 0.13 |
| *CASC1* | 0.13 |
| *DFFA* | 0.13 |
| *C8ORFK32* | 0.13 |
| *FLJ40125* | 0.13 |
| *SLC7A9* | 0.13 |
| *LOC728780* | 0.13 |
| *SEMA6C* | 0.13 |
| *NBR1* | 0.13 |
| *KIAA1715* | 0.13 |
| *FUT1* | 0.13 |
| *ADD3* | 0.13 |
| *ZNF462* | 0.13 |
| *SLC35F5* | 0.13 |
| *STK17A* | 0.13 |
| *PANX2* | 0.13 |
| *RGS3* | 0.13 |
| *PLEKHH1* | 0.13 |
| *CSNK1G1* | 0.13 |
| *UBE2G2* | 0.13 |
| *DCTN1* | 0.13 |
| *GPR113* | 0.13 |
| *ABCA5* | 0.13 |
| *AANAT* | 0.13 |
| *BAIAP2* | 0.13 |
| *LARS* | 0.13 |
| *NPAL3* | 0.13 |
| *AP1GBP1* | 0.13 |
| *MAB21L2* | 0.13 |
| *RFFL* | 0.13 |
| *SYMPK* | 0.13 |
| *C11orf17* | 0.13 |
| *MAPKAPK5* | 0.13 |
| *TTC39B* | 0.13 |
| *SAR1B* | 0.13 |
| *PSME1* | 0.13 |
| *GRHL1* | 0.13 |
| *CEP70* | 0.13 |
| *RBPMS2* | 0.13 |
| *IL27* | 0.13 |
| *DNAH17* | 0.13 |
| *P11* | 0.13 |
| *LILRA4* | 0.13 |
| *HOXC8* | 0.13 |
| *MANBA* | 0.13 |
| *CHMP6* | 0.13 |
| *CDKN2AIPNL* | 0.13 |
| *SULT1A1* | 0.13 |
| *MYSM1* | 0.12 |
| *AKNA* | 0.12 |
| *RTBDN* | 0.12 |
| *UPP1* | 0.12 |
| *ACBD7* | 0.12 |
| *ZDHHC4* | 0.12 |
| *SNX29* | 0.12 |
| *CD59* | 0.12 |
| *RSPRY1* | 0.12 |
| *BRF1* | 0.12 |
| *CLMN* | 0.12 |
| *C11orf1* | 0.12 |
| *PGAM1* | 0.12 |
| *EZH2* | 0.12 |
| *AGRN* | 0.12 |
| *GAS2L2* | 0.12 |
| *STEAP3* | 0.12 |
| *ADAMTS5* | 0.12 |
| *PEAR1* | 0.12 |
| *PTPN18* | 0.12 |
| *TOX4* | 0.12 |
| *AMT* | 0.12 |
| *FAM20C* | 0.12 |
| *CRABP1* | 0.12 |
| *SIGLEC9* | 0.12 |
| *MMP25* | 0.12 |
| *INTS3* | 0.12 |
| *ADH1A* | 0.12 |
| *PCDHGB4* | 0.12 |
| *TRPC3* | 0.12 |
| *POU5F1P1* | 0.12 |
| *RIOK1* | 0.12 |
| *RNH1* | 0.12 |
| *MTSS1L* | 0.12 |
| *SHB* | 0.12 |
| *ABT1* | 0.12 |
| *ANKRD9* | 0.12 |
| *API5* | 0.12 |
| *HELZ* | 0.12 |
| *SLC22A18* | 0.12 |
| *C10orf118* | 0.12 |
| *RHBDD2* | 0.12 |
| *ADSSL1* | 0.12 |
| *FRMD5* | 0.12 |
| *MYST3* | 0.12 |
| *AURKC* | 0.12 |
| *FKBP2* | 0.12 |
| *B3GNTL1* | 0.12 |
| *TMEM17* | 0.12 |
| *ZNF697* | 0.12 |
| *PLA2G4E* | 0.12 |
| *TAS2R43* | 0.12 |
| *WDR1* | 0.12 |
| *EXTL3* | 0.12 |
| *C10orf26* | 0.12 |
| *WBP2NL* | 0.12 |
| *RIC8B* | 0.12 |
| *KCNN2* | 0.12 |
| *SF3B1* | 0.12 |
| *CITED4* | 0.12 |
| *VSX1* | 0.12 |
| *RSHL3* | 0.12 |
| *BAT1* | 0.12 |
| *CRLF1* | 0.12 |
| *ANKRD18A* | 0.12 |
| *FBXO43* | 0.12 |
| *SNRNP48* | 0.12 |
| *SLC6A6* | 0.12 |
| *ATP8B4* | 0.12 |
| *GRN* | 0.12 |
| *MORN1* | 0.12 |
| *DGUOK* | 0.12 |
| *C6orf106* | 0.12 |
| *KIF5C* | 0.12 |
| *C1orf75* | 0.12 |
| *C9orf173* | 0.12 |
| *FLJ30851* | 0.12 |
| *TRIM23* | 0.12 |
| *TRPM3* | 0.12 |
| *SLC38A10* | 0.12 |
| *CNGA3* | 0.12 |
| *PXMP2* | 0.12 |
| *AGFG1* | 0.12 |
| *ARMC2* | 0.12 |
| *INPP4A* | 0.12 |
| *ART5* | 0.12 |
| *RGS19* | 0.12 |
| *NFKBIL2* | 0.12 |
| *SPRN* | 0.12 |
| *C1orf27* | 0.12 |
| *LEKR1* | 0.12 |
| *PASK* | 0.12 |
| *SUOX* | 0.12 |
| *USP53* | 0.11 |
| *QRFP* | 0.11 |
| *WWOX* | 0.11 |
| *DBH* | 0.11 |
| *C19orf40* | 0.11 |
| *RGS11* | 0.11 |
| *MED7* | 0.11 |
| *TUB* | 0.11 |
| *F11* | 0.11 |
| *ATP7B* | 0.11 |
| *PI4KA* | 0.11 |
| *UMODL1* | 0.11 |
| *ATP5G3* | 0.11 |
| *PIAS2* | 0.11 |
| *MRPL14* | 0.11 |
| *NCCRP1* | 0.11 |
| *BMP2K* | 0.11 |
| *FAM48A* | 0.11 |
| *SRGAP3* | 0.11 |
| *HLA_DQA1* | 0.11 |
| *TTC21B* | 0.11 |
| *VWA2* | 0.11 |
| *TAF1C* | 0.11 |
| *KIAA1641* | 0.11 |
| *DCAF10* | 0.11 |
| *RBM8A* | 0.11 |
| *CYP51A1* | 0.11 |
| *LIG3* | 0.11 |
| *ACSL6* | 0.11 |
| *PTK9* | 0.11 |
| *WBSCR17* | 0.11 |
| *NCF1* | 0.11 |
| *ZNF337* | 0.11 |
| *ARID3B* | 0.11 |
| *COL8A2* | 0.11 |
| *ASPSCR1* | 0.11 |
| *PTGES* | 0.11 |
| *FAM21C* | 0.11 |
| *TMEM81* | 0.11 |
| *OSBPL5* | 0.11 |
| *PLEKHA2* | 0.11 |
| *FLJ40411* | 0.11 |
| *KANK1* | 0.11 |
| *C5orf40* | 0.11 |
| *ATG7* | 0.11 |
| *FBXO42* | 0.11 |
| *CPZ* | 0.11 |
| *B4GALNT1* | 0.11 |
| *CLEC1A* | 0.11 |
| *TXNDC11* | 0.11 |
| *CCM2* | 0.11 |
| *NMUR1* | 0.11 |
| *SEPT12* | 0.11 |
| *SCGBL* | 0.11 |
| *HEATR3* | 0.11 |
| *PIGO* | 0.11 |
| *MAST2* | 0.11 |
| *AGER* | 0.11 |
| *ASAH1* | 0.11 |
| *BAG5* | 0.11 |
| *KIAA1147* | 0.11 |
| *SPTLC3* | 0.11 |
| *SOAT1* | 0.11 |
| *MAN1A2* | 0.11 |
| *PHF11* | 0.11 |
| *LRP1* | 0.11 |
| *DSG3* | 0.11 |
| *C16orf73* | 0.11 |
| *RIPK2* | 0.11 |
| *EME2* | 0.11 |
| *KCNK10* | 0.11 |
| *HSPA12A* | 0.11 |
| *GALNTL1* | 0.11 |
| *CUGBP2* | 0.11 |
| *SLC17A5* | 0.11 |
| *LSM14A* | 0.11 |
| *TGM1* | 0.11 |
| *ACADSB* | 0.11 |
| *SMN1* | 0.11 |
| *ECHDC2* | 0.11 |
| *C1orf58* | 0.11 |
| *C5orf37* | 0.11 |
| *ASCL5* | 0.11 |
| *SPG21* | 0.11 |
| *ZNF468* | 0.11 |
| *PKD1L1* | 0.11 |
| *IGSF22* | 0.11 |
| *KIAA0467* | 0.11 |
| *TAF11* | 0.11 |
| *CXCL12* | 0.11 |
| *CALB2* | 0.11 |
| *POP5* | 0.11 |
| *FAM43B* | 0.11 |
| *CTU2* | 0.11 |
| *PPIL5* | 0.11 |
| *DCP1B* | 0.11 |
| *SPCS3* | 0.11 |
| *TIGD1* | 0.11 |
| *ZNF550* | 0.11 |
| *EXT2* | 0.11 |
| *KIF6* | 0.11 |
| *BANP* | 0.11 |
| *MCM8* | 0.11 |
| *LRRC46* | 0.11 |
| *TMC8* | 0.11 |
| *GUCA1B* | 0.11 |
| *SEPT1* | 0.11 |
| *CYP21A2* | 0.11 |
| *TAOK2* | 0.11 |
| *RPL18A* | 0.11 |
| *KIAA1370* | 0.11 |
| *RELT* | 0.11 |
| *CEP63* | 0.11 |
| *FGD2* | 0.11 |
| *ALG11* | 0.11 |
| *C14orf53* | 0.11 |
| *TSSK3* | 0.11 |
| *CATSPER2* | 0.11 |
| *MMP16* | 0.11 |
| *PPA2* | 0.11 |
| *CHI3L2* | 0.11 |
| *ADCY2* | 0.11 |
| *APTX* | 0.10 |
| *EEF2K* | 0.10 |
| *SPAG1* | 0.10 |
| *NACAD* | 0.10 |
| *TXNDC14* | 0.10 |
| *PGD* | 0.10 |
| *NUDT9* | 0.10 |
| *SLC2A12* | 0.10 |
| *ORC4L* | 0.10 |
| *NIPAL4* | 0.10 |
| *FAM126A* | 0.10 |
| *RPF2* | 0.10 |
| *SRI* | 0.10 |
| *C1QTNF9B* | 0.10 |
| *ARMS2* | 0.10 |
| *CAPN11* | 0.10 |
| *STIM2* | 0.10 |
| *ASB16* | 0.10 |
| *IFI35* | 0.10 |
| *ZNF16* | 0.10 |
| *PPP6C* | 0.10 |
| *CCDC101* | 0.10 |
| *GPX1* | 0.10 |
| *C5orf54* | 0.10 |
| *DPM2* | 0.10 |
| *EPS8L1* | 0.10 |
| *LPPR1* | 0.10 |
| *NFATC2IP* | 0.10 |
| *KIF1B* | 0.10 |
| *FAM3C* | 0.10 |
| *PON1* | 0.10 |
| *WSCD2* | 0.10 |
| *ASCC2* | 0.10 |
| *ZNF229* | 0.10 |
| *AGAP6* | 0.10 |
| *PDCD6IP* | 0.10 |
| *INADL* | 0.10 |
| *TPPP2* | 0.10 |
| *DMRT2* | 0.10 |
| *PARD3* | 0.10 |
| *SLC41A3* | 0.10 |
| *GRIA4* | 0.10 |
| *CAPNS2* | 0.10 |
| *ABCB9* | 0.10 |
| *B3GNT1* | 0.10 |
| *PIGL* | 0.10 |
| *C1orf114* | 0.10 |
| *SPATC1* | 0.10 |
| *STYX* | 0.10 |
| *CAV2* | 0.10 |
| *SLC5A4* | 0.10 |
