# Supplementary material for: Genotype-Based Gene Expression in Colon Tissue—Prediction Accuracy and Relationship with the Prognosis of Colorectal Cancer Patients
Source: Int J Mol Sci. 2020 Oct 31;21(21):8150. doi: 10.3390/ijms21218150 (PMC7662650; doi:10.3390/ijms21218150)
Supplement: Supplementary file 1 [file ijms-21-08150-s001.zip › Supplementary Material/TableS3.docx]

**Table S3:** SNPs and their corresponding weights used for the prediction of the genes *TRIM4* and *PYGL* (extracted from the PrediXcan transverse colon prediction model).

| Gene | SNP | Weight |
| --- | --- | --- |
| *TRIM4* | rs1025576 | 0.0067 |
| *TRIM4* | rs1048705 | 0.0282 |
| *TRIM4* | rs10808114 | 0.0270 |
| *TRIM4* | rs2082744 | 0.0289 |
| *TRIM4* | rs2247761 | 0.0268 |
| *TRIM4* | rs2293768 | 0.0287 |
| *TRIM4* | rs2525550 | 0.0380 |
| *TRIM4* | rs2527899 | 0.0566 |
| *TRIM4* | rs2527919 | 0.0271 |
| *TRIM4* | rs2527922 | 0.0276 |
| *TRIM4* | rs2572003 | 0.0266 |
| *TRIM4* | rs2572005 | 0.0273 |
| *TRIM4* | rs2572006 | 0.0274 |
| *TRIM4* | rs2572008 | 0.0278 |
| *TRIM4* | rs2572009 | 0.0280 |
| *TRIM4* | rs2572010 | 0.0286 |
| *TRIM4* | rs2572019 | 0.0290 |
| *TRIM4* | rs2572022 | 0.0072 |
| *TRIM4* | rs4236540 | 0.0006 |
| *TRIM4* | rs472660 | 0.0462 |
| *TRIM4* | rs474229 | 0.0053 |
| *PYGL* | rs17662696 | 0.0079 |
| *PYGL* | rs1890700 | -0.0141 |
| *PYGL* | rs1890706 | -0.0371 |
| *PYGL* | rs1951473 | 0.0074 |
| *PYGL* | rs1956568 | -0.0470 |
| *PYGL* | rs1983733 | -0.0141 |
| *PYGL* | rs2181083 | 0.0298 |
| *PYGL* | rs2983088 | -0.0071 |
| *PYGL* | rs3783273 | -0.0245 |
| *PYGL* | rs3783274 | -0.0096 |
| *PYGL* | rs3825541 | -0.0245 |
| *PYGL* | rs4480710 | -0.0011 |
| *PYGL* | rs4608255 | -0.0003 |
| *PYGL* | rs4901066 | -0.0143 |
| *PYGL* | rs6572791 | -0.0058 |
| *PYGL* | rs6572792 | -0.0034 |
| *PYGL* | rs7146474 | -0.0076 |
| *PYGL* | rs7149295 | -0.0124 |
| *PYGL* | rs7152773 | 0.0041 |
| *PYGL* | rs7159833 | -0.0085 |
| *PYGL* | rs720198 | -0.0121 |
| *PYGL* | rs8010174 | -0.0017 |
| *PYGL* | rs8013076 | -0.0060 |
| *PYGL* | rs8014291 | -0.0308 |
| *PYGL* | rs8018152 | 0.0874 |
| *PYGL* | rs885251 | 0.0023 |
| *PYGL* | rs885375 | -0.1162 |
