# Supplementary material for: Genotype-Based Gene Expression in Colon Tissue—Prediction Accuracy and Relationship with the Prognosis of Colorectal Cancer Patients
Source: Int J Mol Sci. 2020 Oct 31;21(21):8150. doi: 10.3390/ijms21218150 (PMC7662650; doi:10.3390/ijms21218150)
Supplement: Supplementary file 1 [file ijms-21-08150-s001.zip › Supplementary Material/TableS5.docx]

**Table S5:** Characteristics of the 4241 colorectal cancer patients from the validation set.

| **Variable** | **Level** | **Patients** |
| --- | --- | --- |
| Age at diagnosis (years) | < 60 | 1579 |
|  | 60 – 69 | 1986 |
|  | 70 – 79 | 676 |
|  | ≥ 80 | 0 |
| Gender | Male | 2443 |
|  | Female | 1798 |
| Body mass index (kg/m^2^) | < 18.5 | 14 |
|  | 18.5-24.9 | 1171 |
|  | 25-29.9 | 1918 |
|  | ≥ 30 | 1115 |
| Diabetes | No | 3892 |
|  | Yes | 337 |
| Smoking | Never | 411 |
|  | Former | 382 |
|  | Current | 17 |
