# Supplementary figures and images for: Genotype-Based Gene Expression in Colon Tissue—Prediction Accuracy and Relationship with the Prognosis of Colorectal Cancer Patients
Source: Int J Mol Sci. 2020 Oct 31;21(21):8150. doi: 10.3390/ijms21218150 (PMC7662650; doi:10.3390/ijms21218150)

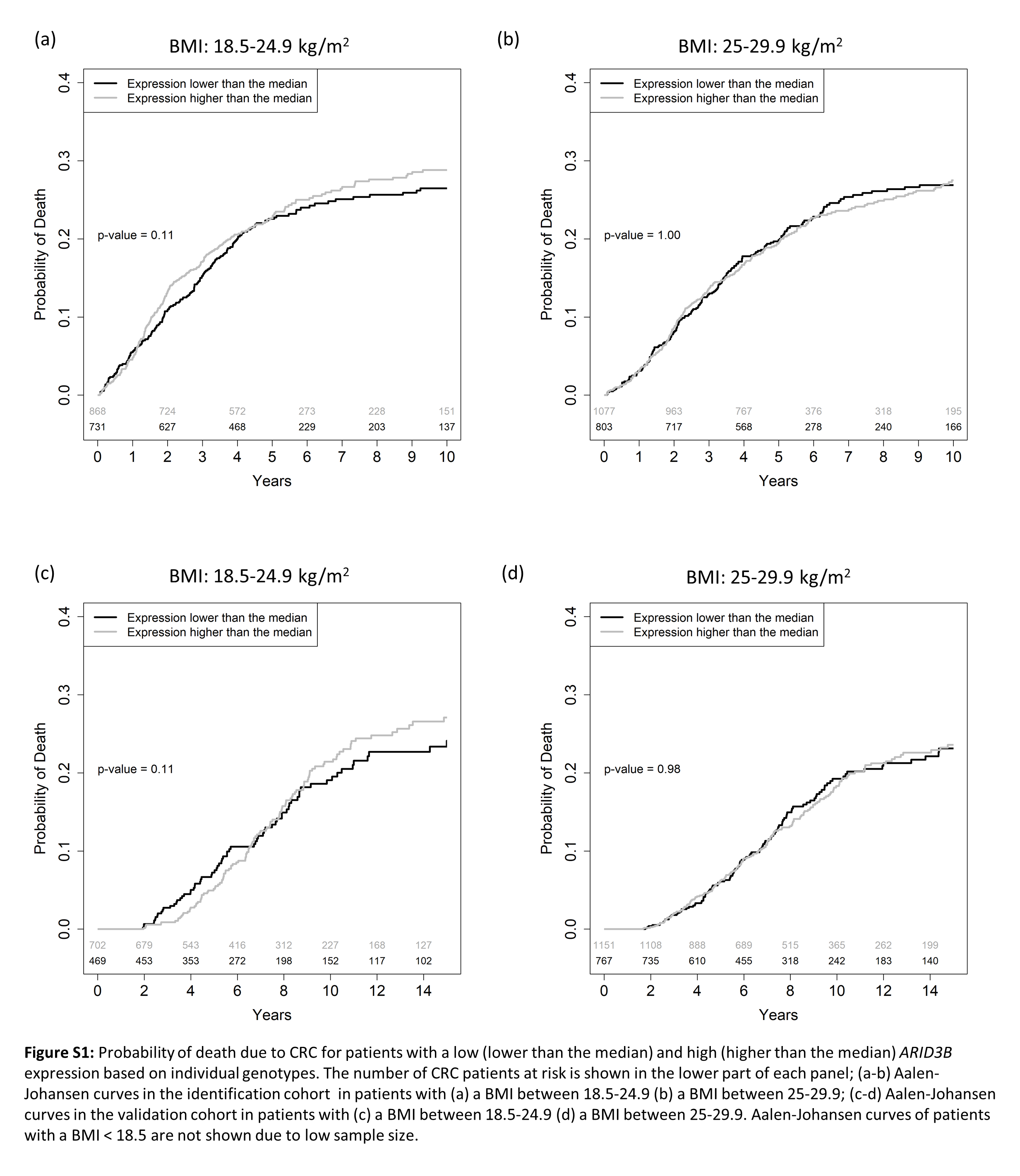

Supplement: Supplementary file 1 [file ijms-21-08150-s001.zip › Supplementary Material/FigureS1.tif]
